# Effects of Physical Stimulation in the Field of Oral Health

**DOI:** 10.1155/2021/5517567

**Published:** 2021-04-07

**Authors:** Yanxin Qi, ShuXin Zhang, Mi Zhang, Zili Zhou, Xinyi Zhang, Wenhui Li, HongXin Cai, Bing Cheng Zhao, Eui-Seok Lee, Heng Bo Jiang

**Affiliations:** ^1^Stomatological Materials Laboratory, School of Stomatology, Shandong First Medical University & Shandong Academy of Medical Sciences, Tai'an, Shandong 271016, China; ^2^Department of Oral and Maxillofacial Surgery, Graduate School of Clinical Dentistry, Korea University, Seoul 08308, Republic of Korea

## Abstract

Physical stimulation has been widely used in clinical medicine and healthcare due to its noninvasiveness. The main applications of physical stimulation in the oral cavity include laser, ultrasound, magnetic field, and vibration, which have photothermal, cavitation, magnetocaloric, and mechanical effects, respectively. In addition, the above four stimulations with their unique biological effects, which can play a role at the gene, protein, and cell levels, can provide new methods for the treatment and prevention of common oral diseases. These four physical stimulations have been used as important auxiliary treatment methods in the field of orthodontics, implants, periodontal, dental pulp, maxillofacial surgery, and oral mucosa. This paper systematically describes the application of physical stimulation as a therapeutic method in the field of stomatology to provide guidance for clinicians. In addition, some applications of physical stimulation in specific directions are still at the research stage, and the specific mechanism has not been fully elucidated. To encourage further research on the oral applications of physical stimulation, we elaborate the research results and development history of various physical stimuli in the field of oral health.

## 1. Introduction

Currently, the development of clinical therapies is mainly based on chemical achievements, and chemical achievements have been further utilized and expanded in the pharmaceutical industry. However, most drugs do not only affect the target tissue, but also affect the entire body, causing side effects in many cases. In contrast, general physical medicine, such as sound waves, magnetic fields, lasers, and mechanical vibrations, provides a noninvasive, safe, and easy-to-apply method to directly act on the injury site and to control the source of pain and inflammation [1]. Different physical stimulations have different effects on tissues and cells, although it can generally reduce inflammation, relieve pain, and improve immune function. It is a good mean to promote, to maintain, and to restore various functions of the human body [2].

In the oral cavity, treatment usually involves surgical procedures and drug therapy. Traditional surgery is the main method to treat oral diseases, although it requires strict indications and can cause trauma during treatment. Common surgical procedures such as root canal treatment, orthodontic treatment, and implants have problems such as incomplete root canal irrigation, long course of orthodontic treatment accompanied by pain, and an excessive implant healing cycle, respectively. In addition, drug therapy has always been the main method for the treatment of mucosal diseases, which is often universal and nonspecific, and can easily lead to bacterial resistance. With the intersection of physical therapy and the oral field, physical therapy is gradually applied to assist clinical treatment to achieve the purpose of reducing pain, thereby promoting healing, accelerating bone remodeling, and inhibiting inflammation and bacterial reproduction. This paper mainly describes the clinical application and development process of four kinds of physical stimulation in implant, orthodontic, dental pulp, periodontal, prosthodontic, mucosal, and maxillofacial surgery and describes its influence on oral treatment at the cellular, tissue, and individual levels.

## 2. Introduction to Physical Stimulation

### 2.1. Laser

Laser is the stimulated emission of light generated by a large number of particles excited by the coherent radiation field. According to the transmission form and wavelength, lasers can be divided into carbon dioxide (CO_2_), Nd: YAG, Er: YAG, Er Cr: YSGG, diode lasers, and others. In the process of orthodontic treatment, it can accelerate tooth movement and reduce pain and periodontal inflammation during orthodontic treatment. In terms of implants, laser can improve the initial stability of implants and treat peri-implantitis. Besides, laser can be used to remove dental calculus and dental plaque, to promote wound healing and bone healing, and to remove hyperplastic gums in periodontal treatment. In terms of dental pulp, laser can be used for the treatment of dentin hypersensitivity and dental caries, and can also be used to assist root canal treatment. In addition, laser is used for the treatment of oral mucosal inflammation. It can reduce pain, promote wound healing, and shorten recovery time.

### 2.2. Magnetic Field

The magnetic field is generated by the moving charge or changing electric field and refers to the field that transmits the magnetic force between objects. Electromagnetic technology was developed by Maxwell in 1865, although in the 1980s, its role as a treatment attracted the interest of basic scientists and clinicians. According to the different generation methods, the magnetic field used in the oral cavity is mainly divided into pulsed electromagnetic field and static magnetic field generated by magnetic nanoparticles or magnetic materials. Currently, the magnetic field is used to regenerate the pulp and dentin, to promote mandibular fracture repair and implant bone healing, to shorten the orthodontic treatment course, and to relieve pain during the orthodontic process, and its magnetocaloric effect is used to target the oral mucosal squamous cell carcinoma.

### 2.3. Ultrasound

Ultrasound, as a form of high-frequency acoustic wave propagating energy through biological tissues, has been applied in several medical fields, and the safety of ultrasound used in long-term treatment has been improved. In the field of stomatology, ultrasonic stimulation is used for physical therapy, which can play an important role in periodontal, orthodontic, implant, and oral preventive health care. Ultrasound can eliminate dental plaque and calculus in periodontal tissues through the cavitation effect and acoustic microstreaming to achieve the anti-inflammatory, cleaning, and sterilization effect. In addition, the biological effect of ultrasound can promote bone remodeling to further accelerate the orthodontic movement speed and improve the survival rate and stability of implants by promoting bone healing. As a noninvasive technology, low-intensity pulsed ultrasound (LIPUS) is a new kind of physical stimulation that can help to treat injuries by emitting sound waves. It has been shown to play an important role in cell metabolism and tissue repair [3] and is widely used in medicine. In the dental field, LIPUS has entered the clinical stage in root canal flushing and plaque removal.

### 2.4. Mechanical Vibration

Vibration applied in stomatology is aimed at promoting bone remodeling in clinical practice, preventing osteoporosis, and accelerating orthodontic treatment. However, vibration can not only affect the alveolar bone but also affect the restoration of other periodontal tissues. Studies have shown that mechanical stress affects the cell shape and cytoskeletal structure, controlling many cellular behaviors critical to tissue development, including migration, growth, differentiation, apoptosis, and stem cell lineage transformation [4]. In clinical practice, vibration, as a noninvasive and physical means, has been applied to bone regeneration, joint ligament repair, and in the oral cavity and other fields [5]. Whole-body vibration has been shown to prevent osteoporosis and bone loss and to promote muscle formation. In orthodontic treatment, Mani et al. found that vibration can accelerate tooth movement and reduce postorthodontic pain by promoting fibroblast repair and affecting bone tissue reconstruction [6, 7].

## 3. Application of Laser in the Oral Cavity

### 3.1. Shortened Orthodontic Treatment Course and Orthodontic Pain Relief

The therapeutic effects of laser on orthodontics mainly include three aspects: shortening the course of orthodontic treatment, reducing pain, and relieving periodontal inflammation caused by orthodontics. Delma et al. used GaAlAs lasers to conduct experiments on orthodontic patients and found that low-energy laser technology could greatly shorten the time of orthodontic treatment without damaging teeth and periodontal tissues [8]. The specific mechanisms include promoting vascular regeneration to provide nutrition for periodontal tissue reconstruction and promoting the proliferation of osteoclasts and periodontal ligament cells and formation of mineralized bone, which promote the reconstruction of periodontal tissue and accelerate tooth movement [9]. Ren et al. used 940 nm diodes (EZlase; Biolase Technology Inc.) to treat orthodontic patients and found that it can control the inflammation of periodontal tissue during orthodontic treatment. It controls the expression of factors such as cyclooxygenase-2, interleukin-1*β* (IL-*β*), platelet-derived growth factor, transforming growth factor-*β*, basic fibroblast growth factor, and other factors to control the orthodontic treatment of middle teeth inflammation of peripheral tissues. Besides, laser can reduce pain by reducing the increased levels of IL-*β*, prostaglandin E2 (PGE2), substance P, and other pain mediators; the patients' VAS score also confirmed this conclusion [10].

### 3.2. Improvement in the Success Rate of Dental Implants

Laser was first used in the second-stage exposure surgery of implants because of its good bleeding control effect, precise cutting effect, and local disinfection [11]. In addition, low-energy laser irradiation can promote osseointegration, improve the secondary stability of implants, and treat peri-implantitis; therefore, it is widely used in the field of oral implantation. Animal and cell experiments have showed that low-intensity laser irradiation can promote the proliferation and differentiation of osteoblasts by promoting the expression of BMP-2. Laser plays a role in accelerating bone formation around the implant [11, 12] and promoting the stability of the implant [13]. In clinical trials, Mandić et al. performed low-intensity laser irradiation on the posterior maxillary teeth of 12 patients, and from Table 1, we found that the irradiated implant had higher stability, and the difference reached a significant level at the 5^th^ week after surgery [14].

Additionally, low-level laser therapy (LLLT) can also treat peri-implantitis, which is considered to be one of the main causes of implant failure. The accumulation of plaque on the surface of the implant is considered to be the initiating factor leading to the peri-implant inflammation. Bacteria can inhibit the growth of bone cells and stimulate osteoclasts to cause bone resorption. In Takasaki-treated dogs with Er: YAG laser-induced inflammation around the implant, good new bone formation was found on the surface of the implant and the implant-bone contact area increased [15]. Clinically, patients with peri-implantitis were irradiated with Er: YAG laser, and the plaque index, bleeding on probing, and gingival recession were all improved, which proved that the laser can promote bone formation and inhibit inflammation around implants to improve the success rate of the implant effectively [16].

Comparative analysis between the groups showed that the ISQ value of the experimental group was higher than that of the control group during the entire 6-week observation period, and the difference was statistically significant in the 5th week [14].

### 3.3. Treatment of Periodontal Disease

In terms of periodontal disease, laser can be used to remove dental calculus and plaque, to promote wound healing, and to promote the repair of bone defects. In addition, laser can also remove hyperplastic gums, to shorten the course of orthodontic treatment, and to treat orthodontic pain and oral mucosal inflammation.

### 3.4. Removal of Dental Calculus and Plaque

Dental calculus is an important pathogenic factor in the development of periodontal disease. After the calculus forms, it can compress the gums and affect blood circulation; besides, calculus contains a lot of water and inorganic substances, which is conducive to the growth of anaerobic bacteria, can easily cause bacterial infection of periodontal tissues, and form periodontal pockets. Therefore, removing calculus is the most critical step in the treatment of periodontitis. Er: YAG laser has good water absorbability. It can evaporate the water in the calculus to increase the pressure in the calculus, cause the calculus to burst, and remove the calculus. Clinically, 30 untreated mesial and distal tooth surfaces were treated with hand instruments and Er: YAG laser irradiation, respectively, which proved that the Er: YAG laser has the ability to remove calculus. Although its effectiveness is lower than mechanical cleaning, it can be improved by extending the course of treatment. Moreover, laser treatment has a better effect on cement preservation than handheld device treatment [17].

Dental plaque is an important factor in the occurrence and aggravation of periodontitis; thus, it is particularly important to control and to treat dental plaque. The most commonly used method now is mechanical treatment, although mechanical treatment is difficult to reach in areas such as the bifurcation of the roots and deep gingival sulcus in patients with periodontal disease, resulting in the recurrence of the disease. Drugs such as antibiotics alone usually cannot effectively remove dental plaque biofilm. Lasers can kill microorganisms on dental plaque, improve the environment of the root part, and facilitate the attachment and growth of periodontal ligament cells on cementum [18]. Reznick et al. showed that the shockwave technology generated by lasers can effectively disrupt *P. aeruginosa* biofilms *in vitro*, which changes the membrane permeability and eventually allows the antibiotic to penetrate into the bacterial cell and kill it. Therefore, the combination of lasers and antibiotics can result in synergies and then shows a better efficacy against *P. aeruginosa* [19]. Clinical studies have performed laser treatment on patients and found that laser has a significant therapeutic effect on patients' dental plaque and can reduce discomfort during treatment [20, 21].

### 3.5. Promotion of Wound Healing

Wound healing can be divided into three stages: inflammation, proliferation, and reconstruction [22]. Factors affecting wound healing include the proliferation of fibroblasts, collagen synthesis, macrophage stimulation, and extracellular matrix production. Pourzarandian et al. used pulsed Er: YAG lasers with different energy densities to irradiate cultured human gingival fibroblasts and found that the cell growth rate can be accelerated and that the optimal stimulation energy density was 3.37 J/cm^2^ [23]. Laser promotes collagen synthesis in fibroblast cells by accelerating mRNA transcription rate of collagen gene and enhancing the activity of related enzymes [24]. Silveira et al. irradiated rats of the control group with laser and found that the laser could accelerate the wound healing process and promote the synthesis of collagen [25].

### 3.6. Promotion of Bone Defect Repair

**Figure 1:**
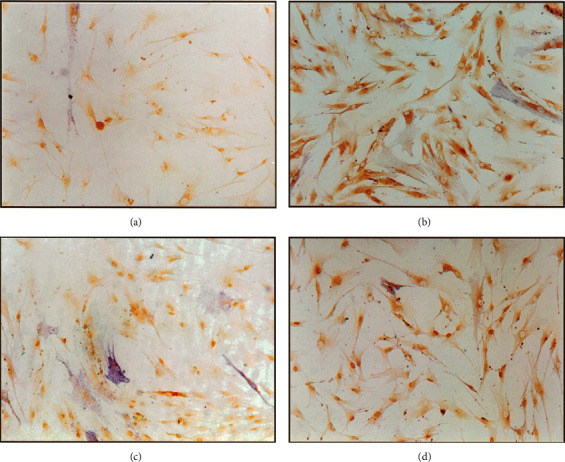
Light microscope micrograph of osteopontin expression in cultured human osteoblast cells 24 h (a) control nonirradiated cells and (b) laser irradiated cells and 48 h (c) control nonirradiated cells and (d) laser irradiated cells postsecond irradiation. Note very light positive staining in the control cells as compared with the significantly stronger positive staining in the irradiated cells (×200). The results showed that the expression of osteopontin in the irradiated cells was significantly increased compared with the unirradiated cells. Osteopontin is one of the osteogenic markers. This shows that laser irradiation in this experiment can promote the proliferation and maturation of human osteoblasts [30].

**Figure 2:**
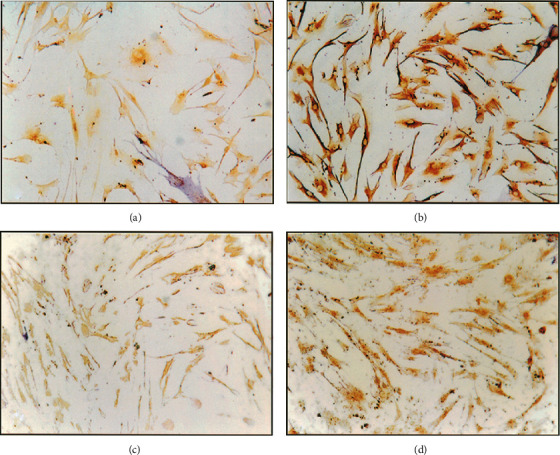
Light microscope micrograph of bone sialoprotein expression in cultured human osteoblast cells 24 h (a) control nonirradiated cells and (b) laser irradiated cells and 48 h (c) control nonirradiated cells and (d) laser irradiated cells postsecond irradiation. Note very light positive staining in the control cells as compared to the significantly stronger staining in the irradiated cells (×200). The results showed that, compared with the nonirradiated cells, bone sialoprotein expression in cells after irradiation significantly increased, and bone sialoprotein is a bone marker into one. Described laser irradiation may facilitate this human osteoblast proliferation and maturation experiment [30].

Due to the fact that bone self-repair is slow, many people have done a lot of research on how to speed up bone healing. One of the most effective nonsurgical methods is LLLT. Cell studies have shown that low-level laser irradiation can enhance the activity of fibroblasts and osteoblasts and promote tissue healing, collagen metabolism, and granulation tissue formation [26, 27]. The possible mechanism of action is that laser stimulates the photoreceptors in the mitochondrial respiratory chain, converting light energy into chemical energy, and changes cellular ATP or cAMP levels [28]. Studies have used LLLT to irradiate the extraction socket of rats. After a period of time, it was found that Runx2, type 1 collagen, osteocalcin, platelet-derived growth factor-B, vascular endothelial growth factor, and other factors related to bone growth all increased [29]. Stein et al. used the He-Ne laser (632 nm) to irradiate human osteoblasts *in vitro* and found that the survival rate of osteoblasts increased, and from Figures 1 and 2, we can see that osteogenic markers increased significantly [30]. Korany et al. irradiated rats with a 75 MW, 830 nm GaAlAs laser and found that the percentage of trabecular bone increased [31]. Therefore, LLLT can promote the healing of tooth extraction sockets, and this effect has been confirmed even in ovariectomized and diabetic rats [32, 33]. In addition, LLLT plays an important role not only for extraction sockets but also in rat mandibular defects [31]. Pretel et al. used 14 J/cm^2^ GaAlAs laser (35 MW, 780 nm) to irradiate the mandibular defect in rats and found that the formation of new bone matrix developed rapidly in the 15-day and 45-day laser application groups [34].

## 4. Removal of Hyperplastic Gum Tissue

Gingival hyperplasia is a common feature of patients with periodontal disease. In clinical practice, gingival resection and angioplasty are usually performed with a scalpel or electric knife, although the comfort level of patients is poor. At present, the laser instruments commonly used for soft tissues include CO_2_ and Nd: YAG lasers. Compared with traditional methods, removing gingival hyperplasia with laser has the advantages of less intraoperative bleeding, faster wound healing, and higher postoperative comfort. It has been applied in the field of clinical medicine [35].

### 4.1. Shortened Orthodontic Treatment Course and Orthodontic Pain Relief

Weichman used high-energy infrared laser to seal the orifice to the root canal for the first time, creating a new field of laser treatment in endodontics [36]. Laser can be used for the treatment of dentine hypersensitivity, dental caries, and root canal treatment. Dentin sensitivity is mainly caused by the wear of the tooth tissue and exposure of the dentin tubules. The external stimulation stimulates the pulp through the dental tubules. The photothermal effect of the laser is used to melt and to recrystallize the dentin surface to seal the dentin tubule opening, thereby isolating the external stimulus. To prevent damage to the root tip, the laser equipment uses low output power and provides enough water to cool the dentin surface and can limit the irradiation time to 10 s to avoid pulp irritation caused by overheating [37]. Kumar et al. confirmed that the Nd: YAG laser had a better effect on sealing dentin tubules than sodium fluoride, and the combination can achieve a better sealing effect [38].

Root canal treatment is the most important method for the treatment of pulp diseases. Bacteria in the root canal is an important pathogenic factor leading to periapical disease of dental pulp. The Nd: YAG laser can produce a photothermal effect; bacteria absorb the heat generated by the laser and then are killed [39, 40]. The laser can dissolve the dentin, and remove the residue and smear layer on the root canal wall. At the same time, the laser can remove the dentin on the root canal wall and enlarge the dentin tubules. Kokuzawa et al. used the Er: YAG laser to irradiate six isolated single human teeth. They found that the smear layer disappeared and the dentin tubules were opened. The Er: YAG laser vaporizes the water in the hard tissue and increases the internal pressure, thus causing the hard tissue to undergo a microexplosion to remove the damaged tissue [41]. Valério et al. treated 29 children with the Er: YAG laser for decayed first molars. It was found that the Er: YAG laser can effectively remove caries tissue and caries causing bacteria such as *Streptococcus mutans* and *Lactobacillus*, and can improve the safety and comfort of surgery [42].

### 4.2. Treatment of Oral Mucosal Inflammation

As a complication of various cancers, oral mucositis seriously affects the patient's treatment enthusiasm and clinical effect. The symptoms include dry mouth, altered taste, burning sensation, and difficulty swallowing. Compared with traditional surgery, laser surgery makes it easier to cut and shape the oral soft tissues, which can reduce bleeding, relieve patient pain, cause less tissue trauma, and heal faster, and is less likely to form scars. A simple laser surgery sometimes only requires surface anesthesia without local infiltration or block anesthesia. Usually, low-intensity laser treatment is used to promote wound healing (LLLT). The photoreceptor on the cell absorbs light energy, promotes the reduction of oxygen by the cytochrome c oxidase on the terminal enzyme of the mitochondrial respiratory chain, increases the synthesis of ATP in the body, and enhances energy metabolism [43]. At present, many experiments have confirmed the promoting effect of laser on the treatment of oral mucositis. Honarmand et al. found that semiconductor laser treatment shortened the recovery time and relieved the pain severity of RHL patients [44]. Sanchez et al. found that compared with acyclovir cream and tablets, laser treatment has fewer side effects and prolongs the recurrence time [45]. Amira et al. conducted follow-up treatment on a young patient with recurrent mucous cysts and also found that the use of 980 nm diode laser treatment can significantly reduce the recurrence rate of mucous cysts. The effect is significant, and diode laser treatment is an efficient, safe, and convenient treatment method [46].

## 5. Application of the Magnetic Field

### 5.1. Promotion of Dental Pulp and Dentin Regeneration

Pulp tissue is susceptible to infection and necrosis, and the pulp tissue after necrosis is difficult to regenerate due to a single blood supply. However, with the development of modern tissue engineering and the discovery of dental stem cells, the regeneration of dental pulp and dentin has been extensively studied [47]. The key to pulp regeneration is the selection of dental pulp stem cells and the establishment of three-dimensional scaffolds. Magnetite nanoparticles (MNPs) are superparamagnetic. The addition of MNPs can improve the mechanical properties of the scaffold and make the material show magnetic [48]. Hyung-mun et al. were the first to use magnetic scaffolds for pulp dentine regeneration. The results showed that magnetic scaffoldings could promote the odontogenic differentiation of dental pulp stem cells by increasing the activity of ALP and mRNA expression of odontogenic markers (DMP-1, DSPP, osteocalcin, and osteocalcin) and enhancing the adhesion and migration of PSCS [49]. These results indicate that magnetic scaffolds can provide good matrix conditions for dentine regeneration.

### 5.2. Treatment and Prevention of Periodontitis

The plaque attached to the gum and tooth surface has been considered as one of the predisposing factors of periodontal disease. Brushing has been widely accepted as the standard hygiene method for controlling scar formation on the gums and has greatly improved the periodontal health, although periodontal disease is still very common and causes huge medical care costs [50]. As a noninvasive physical stimulus, magnetic field can reduce the number of plaque bacteria, enhance the susceptibility of plaque biofilm to antibiotics, and inhibit the formation of plaque biofilm to treat periodontal disease.

Snezana et al. demonstrated the positive effect of magnetic fields on the reduction of plaque bacteria *in vitro*. During the first 24 h of exposure, the number of all isolated microbes was significantly reduced [51]. At present, there is still a controversy about the effect of electromagnetism on oral microbes. Morrow found that it does not have a significant impact on the growth and reproduction of the normal oral flora. Therefore, it is speculated that electromagnetism may only have an effect on disease-curing oral microorganisms, especially those that are involved in the formation of dental plaque. There is almost no effect on the normal oral flora, which further confirms its effectiveness and safety [52]. A reduction in the number of microbes was found under overdentures containing implanted magnets. This plays an important role in protecting the health of the periodontal tissue around the overdenture [53].

With the use of antibiotics, many bacteria have become resistant. As mentioned above, the effectiveness of magnetic fields in inhibiting bacterial proliferation has been confirmed. In addition, others have found that magnetic fields can also be used as an auxiliary means to use MNP/alternating magnetic fields through the magnetocaloric effect to increase part of the temperature, leading to an increase in the metabolic activity of *Staphylococcus aureus*, and promoting the absorption of antibiotics by the bacterial biofilm, thereby increasing the susceptibility of the bacterial biofilm to antibiotics, to reduce the amount of antibiotics and to assist in the purpose of antibiotics to kill resistant bacteria [54].

During the formation of the dental plaque, bacteria reach the tooth surface through electrostatic adsorption, and the magnetic effect may promote the deposition of bacteria and various ions in the dental plaque by reducing the activity of electrostatic ions. Johnson et al. appropriately combined a magnetic device with an oral irrigator to develop a magnetic water irrigator, which was applied to patients with periodontal disease, and found that it appears to greatly reduce the formation of supragingival tartar and its accompanying plaque. It can be reasonably assumed that this type of flushing device will produce great benefits for self-care or sanitation system [55].

### 5.3. Treatment of Oral Mucosal Lesions

Oral squamous cell carcinoma (OSCC) is one of the most common cancers in the world [56]. OSCC patients have a low survival rate and high metastasis rate. At present, the traditional treatment has not improved the prognosis in general, and the development of targeted therapy provides a new treatment for OSCC. OSCC magnetic nanomaterial-targeted treatment method involves coupling magnetic nanoparticles with antibodies targeting integrin (v*αβ*6). Due to the high expression of V 6 in SCC, the targeted localization of tumor cells can be realized. The heating properties of magnetic nanoparticles were used to induce thermal ablation of tumor cells in a changing magnetic field [57]. Magnetic nanomaterials have a good prospect of targeted therapy in the treatment of OSCC.

### 5.4. Production of Orthodontic Force and Shortened Orthodontic Treatment Course

Clinically, a long orthodontic time can cause a series of problems, such as dental caries, periodontal disease root resorption, and other problems, and increase the pain of patients; therefore, it is necessary to accelerate the orthodontic movement and shorten the orthodontic time [58].

**Figure 3:**
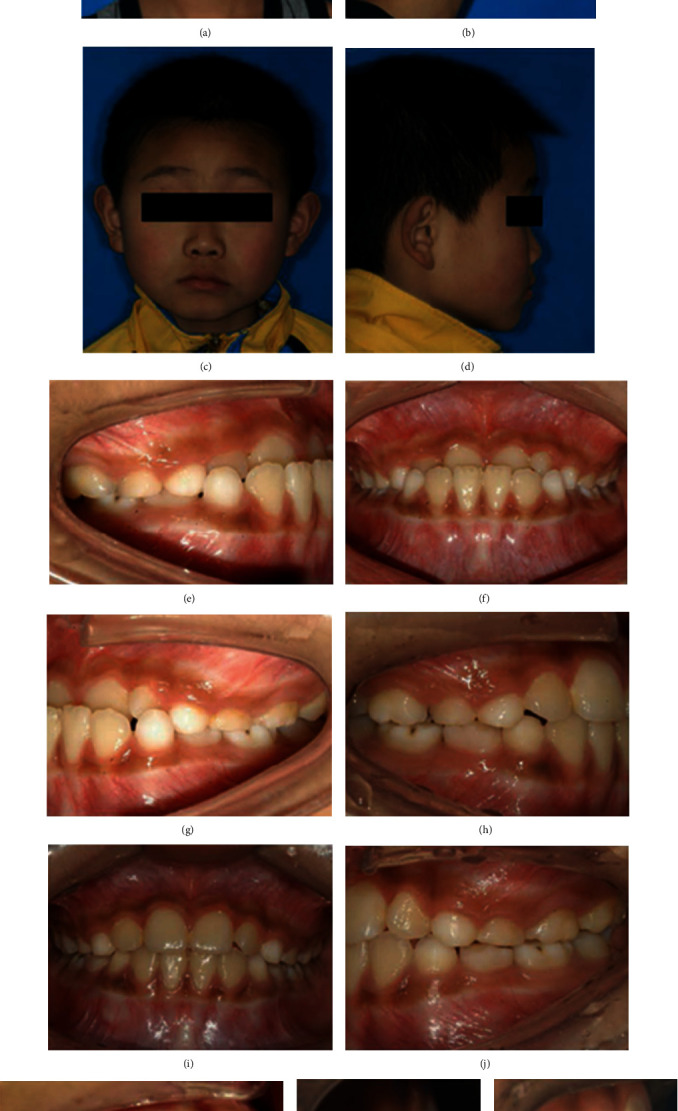
A case treated with MOA-III (comparison of pre- and posttreatment). Pretreatment: extraoral photos (a, b), intraoral photos (e–g), overbite/overjet (l). Posttreatment: extraoral photos (c, d), intraoral photos (h–j), overbite/overjet (m). Intreatment: intraoral photos (k) [62].

At present, the orthodontic devices used in clinical practice cause oral friction to patients, increase the probability of mucosal disease, and aggravate the pain of patients. In addition, the traditional orthodontia cannot significantly improve the malocclusion; therefore, it is very necessary to develop a new orthodontic device. Currently, magnetic methods are used to treat maxillary teeth and class II and class III malocclusions. Compared with the traditional force transfer system, magnets have the following advantages: frictionless mechanics. When the magnet is in the state of attraction, its attraction is controllable and does not decay with time [2]. Animal experiments have shown that the application of 50 Hz ElF-EMF accelerates the movement of orthodontic teeth in rats [59]. In 1977, Kawata et al. designed the first ferrocobalt and chromium magnetic support, although the strength was insufficient and was subsequently replaced by a rare earth magnet, which produced enough orthodontic force. Rare earth magnets can generate a constant magnetic field in the mouth [60, 61]. Studies have shown that EMFs can regulate the proliferation and differentiation of osteoblasts by affecting cell metabolism, changing the structure and morphology of cytoskeleton, and play a role in promoting bone healing, accelerating bone formation, and further accelerating the remodeling of bone tissue associated with orthodontics. Zhao et al. developed a magnetic orthopedic appliance (MOA-III) and applied it to the children with class III malocclusions and found that it could correct the maxilla and mandible at the same time; Figure 3 shows that MOA-III had a good effect on moderate class III malocclusion [62]. In addition, magnets have obvious advantages in guiding the eruption of impacted teeth, such as less stimulation on palatal mucosa, stronger force control, and lower requirements on patient cooperation. These factors may be the main positive characteristics of this alternative therapy.

### 5.5. Improvement in the Implant Success Rate

Implants have become a solution to the epidemic of missing teeth with a high success rate [63]. The factors influencing the success of implants include the initial stability of the implant, bonding between the implant and bone, and number and density of the remaining alveolar bone. One of the key steps in implant healing is to establish osseous healing and osseointegration with the surrounding alveolar bone. Therefore, a variety of treatment methods were proposed to enhance and shorten the time of osseointegration. Pulsed magnetic field has been widely used in clinics to promote bone regeneration. The main principle of pulsed magnetic field is to affect cell differentiation and proliferation by affecting various metabolic pathways, promote angiogenesis and bone tissue formation, and thus promote fracture healing [2, 64]. PEMF was first applied to the oral implants of rabbits, and it was found that the amount of early osseointegration on the implants increased by three times, which verified the effectiveness of PEMF applied to implants for the first time [65]. A miniaturized electromagnetic device that can independently generate a magnetic field was developed to replace the standard healing abutment. It is equipped with a microelectronic module that can generate PEMF to improve bone formation after dental implant surgery. Shlomo et al. applied a miniaturized electromagnetic device (MED) to human oral implants for the first time and found that the initial stability of the implants was significantly improved in the early healing stage [66]. Nayak et al. also found that the average implant stability coefficient (ISQ) of the PEMF group increased by 6.8% in the first two weeks after applying MED, and the overall stability increased by 13%. The inflammatory factor TNF-concentration in the treatment group was significantly decreased in the first four weeks. PEMF stimulation may constitute a valuable new technique for early mastication of implants by promoting bone formation and accelerating bone integration. It can also increase the content of the remaining alveolar bone, thus greatly increasing the success rate of the implant and extending its service life. Some scholars have suggested that PEMF can improve the microenvironment around implants by influencing the level of cytokines [67]. It was shown that PEMF treatment had a positive effect on implant stability by regulating cytokine levels.

### 5.6. Promotion of Mandibular Fracture Healing

The mandible is the second most common site of facial fractures [68, 69]. The most prominent problem in the treatment process is that it requires a long time for fixation, and the subsequent rehabilitation work is delayed. Therefore, shortening the fixation time by accelerating fracture healing has been the subject of research. PEMF has been used as a simple noninvasive method to enhance bone healing. In 1976, Bassett et al. proved for the first time that inductively coupled electromagnetic fields can enhance the effect of bone repair. PEMF stimulation has a beneficial effect on bone healing. The effectiveness of PEMF has been confirmed, although the matrix that promotes bone formation is not fully understood. Some scholars have proposed that PEMF may promote the growth and expansion of blood vessels to promote the healing of tissues at the injured site [2]. Abdelrahim et al. used PEMF for the first time to stimulate patients with mandibular fractures and found that it significantly reduced pain in patients, due to its analgesic and anti-injury effects [70]. Many studies have reported the effectiveness of PEMF in relieving pain. However, Weintraub et al. found that PEMF is not effective in reducing diabetic neuropathic pain. These inconsistent results may be due to the use of fields of different intensities and frequencies [71]. In addition, PEMF reduced early (15 days) bone loss in patients with mandible fracture, and at 30 days postoperatively, possibly with enhanced osteogenesis, bone density was significantly increased in the experimental group [70]. Therefore, as a physical method, PEMF can promote mandibular fracture healing and relieve fracture pain; therefore, it can be used as a clinical auxiliary treatment method and has broad application prospects.

## 6. Application of Ultrasound in the Oral Cavity

### 6.1. Periodontal: Promotion of Periodontal Tissue Regeneration

The purpose of periodontal disease treatment is to prevent periodontal infection and maintain periodontal health. Removing a plaque and calculus is an important treatment and prevention method for periodontal disease. It is believed that the best way of periodontal treatment is to remove the pathogenic bacteria of the periodontal supragingival and subgingival tissues by a nonsurgical machinery. The irritants such as plaque and calculus on the tooth surface can be removed by ultrasound. Cavitation caused by ultrasound ruptures the attached sediments by producing shock waves. In addition, studies have shown that cavitation can enhance the permeability of bacterial cell membrane to antibiotics and can also promote some biochemical reactions in the cell, to be more conductive to the combination of antibiotics and targets and to help antibiotics to kill bacteria [72]. There are two basic types of ultrasonic devices in dentistry: magnetostrictive and piezoelectric devices. Magnetostrictive devices use flat metal strip or metal bars attached to the scaling tip. When the current is provided to the coil, a magnetic field will be generated around the rod sensor, and an alternating magnetic field will be generated by the alternating current, making the tip of the vibrating blade move elliptically. In a piezoelectric element, when an electric current passes through the crystal surface, a change in the size of the crystal contained in the nose reactivates the piezoelectric element, and the resulting vibration leads to a tip motion that is mainly linear in direction and operates differently [73].

As a noninvasive physical stimulation, low-intensity pulsed ultrasound is widely used to promote bone healing and fracture healing by promoting the proliferation of osteoblasts and secretion of angiogenesis-related factors [74].

**Figure 4:**
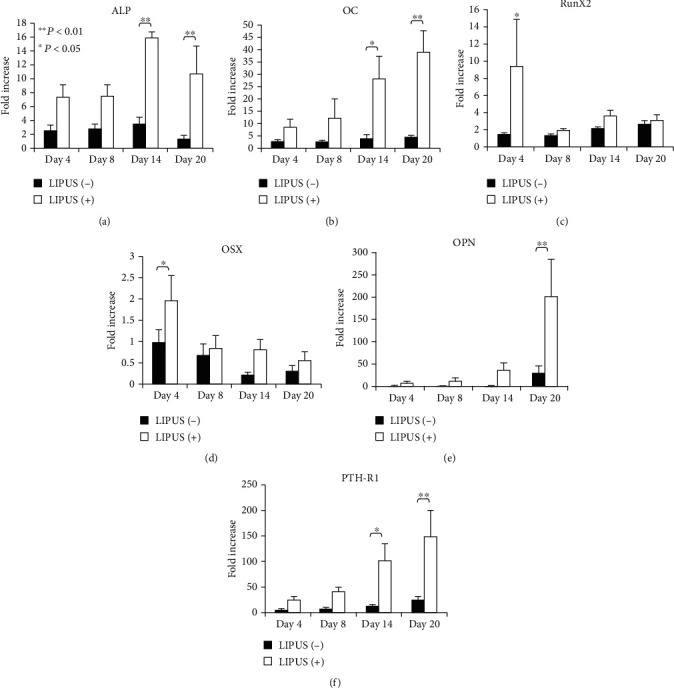
Histogram of real-time PCR gene expression analysis results of 5 cases in the LIPUS(+) group and LIPUS(-) group on the 4th, 8th, and 20th day. The results showed (a) alkaline phosphatase (ALP), (b) osteocalcin (OC), (c) runt-related gene2 (Runx2), (d) osteogenic protein (OSX), (e) bone-bridge Protein (OPN), and (f) parathyroid hormone receptor (PTH-R1). ∗*P* and lt, 0.05; ^∗∗^*P* and lt, 0.01 [77].

In the study of Kitano Lim et al., they used LIPUS to stimulate human alveolar bone-derived mesenchymal stem cells (hABMSCs) derived from the alveolar bone and found that the expression of alkaline phosphatase (ALP) and quantity of cell mineralized nodules was obviously increased after one week, proving that LIPUS promotes the proliferation and osteogenic differentiation of hABMSC [75]. In the experiment of Hu et al., LIPUS stimulation was performed on the periodontal ligament stem cells of adolescent premolars, and it was found that LIPUS could promote the mRNA expression of (ALP), osteocalcin, Runx-2, and integrin B1. Since (ALP) is an important marker of osteogenic differentiation, osteocalcin is used as a late marker of bone formation and osteogenic differentiation. RUNX-2 is an important transcription factor in the osteogenic pathway. Therefore, their study shows that LIPUS can promote the differentiation of bone stromal stem cells and thus enhance periodontal regeneration [76]. Imai et al. applied LIPUS to human mandibular fracture hematoma cells. They detected the expression levels of osteoblast-related gen 2 (Runx2). The expression levels of the osteoblast-related genes ALP, osteocalcin (OC), runt-related gene 2 (Runx2), osterix (OSX), osteopontin (OPN), and parathyroid hormone receptor 1 (PTHR1) were measured by real-time PCR. As shown in Figure 4, the expression levels of ALP, OC, Runx2, OSX, OPN, PTHR1, and mineralization genes were increased in the LIPUS group.

This finding demonstrates the significant influence of LIPUS on MHC osteogenic differentiation and provides important evidence for the potential usefulness of the clinical application of LIPUS in accelerating mandible fracture healing [78].

### 6.2. Improvement in the Implant Success Rate

**Figure 5:**
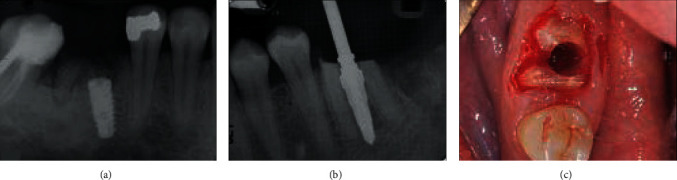
After the initial osteotomy, the implant is placed in the posterior mouth X-ray (a), and the guide pin (bur) is placed during the final osteotomy (b). Use ultrasound equipment in the interradicular bone for final osteotomy preparation (c) [75].

Dental implants have become an important way to repair tooth loss and dentition defect, and it is the first choice for patients with tooth loss. The initial stability of the implant is an important basis for judging the effect of bone healing and determining the repair and loading time, and good initial stability is the key factor affecting the success rate. Scarano offers a way to place implants. Ultrasonic equipment was used to prepare the implant bed before root extraction. As shown in Figure 5, the preparations for osteotomy proceeded normally, and then, the insertion of each ultrasonic inserter was accompanied by osteotomy. It was found that the ISQ level of the control group was 49.9, which was much lower than the experimental group. Compared with traditional preparation, the advantage of this technology is that it is easy to operate and can improve the initial stability of the implant [79]. To shorten the time of bone healing, people have conducted further research on implant osseointegration. Ultrasound has been proven to be an effective method to promote bone healing. Under ultrasonic stimulation, the space between alveolar bone and implant can be filled with new bone trabecula more quickly, achieving the effect of implant binding with surrounding bone. Yakup et al. implanted dental implants into rabbits' femurs and found that the application of LIPUS on oral implants can promote peri-implant osseointegration and increase the bone area at the early stage of osseointegration [80]. Studies have shown that LIPUS can enhance the gene expression of COX-2 and thus enhance the synthesis of endogenous PGE2 in various osteoblastic cell lineages, which is of great significance for bone remodeling [3]. Therefore, ultrasonic stimulation can be an effective method to improve the success rate and stability of oral implants.

### 6.3. Shortened Orthodontic Treatment and Promotion of Root Restoration

**Figure 6:**
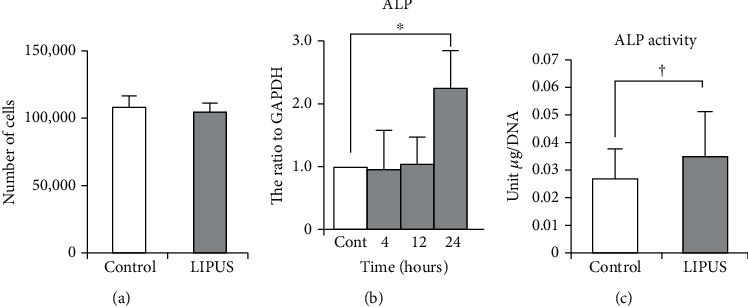
The number of HPL cells (mean ± SD) after being exposed to LIPUS. (a) There was no significant difference in the cell numbers between the control and LIPUS groups (*n* = 5 for each). Effect of LIPUS on the mRNA expression (b) and activity of alkaline phosphatase (c) in HPL cell culture (*n* = 5 for each). Cont: control. ^∗^*P* < 0.01. Values are expressed as mean–SD. ^∗^*P* < 0.01; †*P* < 0.0585.

Orthodontic tooth movement (OTM) is a complex bone remodeling process [81]. Therefore, accelerating alveolar bone remodeling has substantial benefits for patients [82]. Studies have reported that LIPUS promotes alveolar bone remodeling in a rat orthodontic tooth movement model by stimulating the HGF/Runx2/BMP-2 signaling pathway and RANKL expression, further demonstrating that LIPUS stimulation increases Runx2 and BMP-2 expression [83]. During orthodontic treatment, the activity of osteoblasts and osteoclasts around the alveolar bone is highly reactive; therefore, root resorption occurs to a certain extent more or less [84]. Orthodontic-induced inflammatory root resorption is one of the most common side effects of orthodontic treatment. The retention of the tooth body in the alveolar socket is mainly achieved by the cementum of the root, although after the root is damaged, the regeneration ability of the cementum will become very slow and weak. Therefore, a treatment method is needed to restore the damaged root to maintain the integrity of periodontal tissue. Inubushi et al. reported a study to investigate the effects of LIPUS stimulation on the proliferation and differentiation of cementoblast lineage cells. From Figure 6, we can find that compared with the control (untreated cells), LIPUS exposure had no significant effect on the proliferation. The number of cells with LIPUS exposure was almost similar to that in the control. Under the stimulation of LIPUS (*P* < 0.01), the expression level of ALP mRNA within 24 h was significantly increased, and the activity of ALP increased by 1.4 times compared with the control group, which induced the differentiation of human periodontal stem cells into osteoblasts, accelerating the repair of root resorption, and thus enabling the regeneration of periodontal tissue damaged by periodontal disease [85]. The noninvasive nature and safety of LIPUS make it valuable for clinical application [86].

## 7. Application of Mechanical Vibration in the Oral Cavity

### 7.1. The Impact of Vibration on the Periodontal Tissue

Vibration can be used to maintain the health of the periodontium in three aspects: improving the activity of periodontal fibroblasts, promoting the osteogenic differentiation of periodontal stem cells, and maintaining the height of alveolar bone.

As the main component of periodontium, fibroblasts are the main repair cells for soft tissue injuries [87]. They migrate to the site of injury and proliferate, produce large amounts of collagen, and secret a variety of collagen cellulose hormone growth factors. Therefore, Lekic et al. believed that it is the engineer, builder, and manager of trauma repair [88]. Stefan et al. vibrated periodontal membrane fibroblasts through AcceleDent (30 Hz) and VPro5 (120 Hz) and found that both of them increased the secretion of FGF2 and CTGF in fibroblasts. However, FGF2 and CTGF levels after vibration of the VPro5 device were 30-40% higher than those in the AcceleDent group, suggesting that a vibration could promote the activity of fibroblasts, and fibroblast activities were more sensitive to high-frequency vibrations [89].

A large amount of evidence indicates that the best choice for periodontal tissue regeneration is periodontal ligament- (PDL-) derived stem cells/progenitors [90]. Such pluripotent cells can respond to mechanical stimulation, send signals to surrounding cells during orthodontic tooth movement, and play an important role in the regulation of bone reconstruction. Therefore, it is necessary to study the effect of mechanical vibration on PDL. Benjakul S et al. found that mechanical vibration of LMHF promotes the osteogenic differentiation of PDLSC. Studies have shown that after vibration, the expression of PGE2, RANKL, and sRANKL related to osteogenesis and bone resorption role in periodontal ligament stem cells increased, which can promote the process of bone remodeling [91]. Zhang et al. proposed that vibration has a frequency dependent effect in determining the conversion rate of PDLSC to osteoblasts [92]. At the cellular level, a large number of studies have shown that vibration promotes the expression of ALP and type I collagen, which are markers of osteoblast differentiation, and upregulates the level of RUNX2 in osteoclasts and osteoblasts [89]. At the individual level, Alikhani et al. found that after applying a specific frequency 0.3 vibration (peak of acceleration 120 Hz) to the healthy alveolar bone site without stress stimulation of missing teeth but with osteogenesis, alveolar bone structure was preserved after tooth extraction [93, 94]. In the treatment of alveolar bone osteoporosis, Nishimura et al. found that the local application of vibration resulted in increased anabolism and reduced catabolism in alveolar bone in osteoporotic rats [95]. Therefore, vibration has a good prospect in preventing and treating alveolar bone loss caused by tooth loss or osteoporosis.

### 7.2. Effects of Vibration on Orthodontics: Acceleration of the Orthodontic Process and Pain Relief

In recent years, more and more attention has been paid to the application of vibration stimulation in orthodontic treatment. Its purpose is to accelerate the movement speed of orthodontic teeth by accelerating the reconstruction of periodontium and alveolar bone. The research of Nishimura et al. shows that vibration stimulation could accelerate the tooth movement speed of rats and causes no collateral damage to the periodontal tissues [96]. Many studies have reported that a short period of low-intensity high-frequency vibration combined with orthodontic force can increase the frequency of orthodontic tooth movement without causing additional damage to human tissues [97]. One of the studies claimed that a commercially available vibration device could increase the tooth movement distance by 2-3 mm per month and significantly improve the clinical patient acceptance and compliance [5]. Orthodontic force induces pressure on the PDL to cause vascular changes that lead to activation of cellular signaling pathways and the release of proinflammatory molecules such as IL-1 *β*. The survival, fusion, and activation of osteoclasts are related to the level of IL-1*β*. IL-6 regulates alveolar bone remodeling, thereby affecting the amount of tooth movement during orthodontics [97]. Human studies have shown that IL-1*β* level in GCF significantly increases during orthodontic tooth movement. Therefore, vibration can accelerate the reconstruction of periodontal and alveolar bone, thus improving the movement speed of orthodontic teeth [98]. At present, the effectiveness of the vibrator currently used to shorten the course of orthodontic treatment has been confirmed and has been used in clinical practice. During the orthodontic process, due to the force of the tooth, its periodontal tissue will undergo a series of reactions: periodontal tissue is remodeled, and the periodontal ligament is compressed and ischemic, which can lead to the inflammatory response of the periodontal ligament. In addition, improper operation or the friction of the appliance itself on the mucous membrane can cause the patient's pain response. It is believed that temporary tooth displacement can relax the periodontal compression area (including nerve fibers and blocked blood vessels) and thus make the blood flow more easily. In this way, it may prevent the role of pain mediators in this area [99]. Therefore, a mechanical vibrator was proposed to relieve the pain caused by orthodontics. Marie et al. instructed their patients to use the device to vibrate immediately after placing the arch wire, each lasting 15 min, thinking that mechanical vibration may be an effective way to relieve pain [100]. There are few studies on mechanical vibration and the research conclusions are also inconsistent, which may be related to different people's pain sensitivity and different vibration methods. However, there is still a good prospect for improving the pain caused by orthodontic treatment.

## 8. Conclusions

In summary, physical factors play a crucial role in the development and maintenance of many tissues. Living things are always in a constantly changing physical environment and can react to it. Physical stimulation such as laser, magnetic field, sound wave, and vibration play an important role in the treatment and prevention of oral diseases and oral health care. In clinical practice, laser is used in oral soft tissue surgery, such as gingival resection, oral mucosal inflammation treatment, dental caries treatment, root canal preparation and disinfection, and dentin desensitization treatment. As a force source, magnetic field has been widely used in orthodontics and denture retention. Ultrasound can remove dental plaque, promote mandibular fracture healing, and rinse the root canal; vibration massage instrument which can accelerate orthodontic tooth movement has also been widely used in clinic. In addition, physical stimulation has a unique biological effect, which can assist antibiotics to kill oral microorganisms and to maintain oral hygiene. At present, a variety of physical stimulations have been extensively studied in promoting osseointegration of implants, and some of them have entered the stage of clinical experiment. Some of them are still in the research stage, and a large amount of experimental data is still needed to confirm its safety and effectiveness. Physical therapy provides a relatively noninvasive stimulation to promote tissue healing and repair and to relieve the pain of patients compared with the traditional treatment and provides a variety of choices for common oral treatment methods, which is a significant auxiliary means for oral clinicians.

## Figures and Tables

**Table 1:** Differences in implant stability between irradiated (test) and nonirradiated (control) implants.

Time	Implant stability quotient (*x* ± SD)			*P*
	Test	Control	95% CI for MD	
Baseline	76.00 ± 3.52	72.89 ± 7.15	-0.78177 to 7.00399	0.110
1st week	74.88 ± 3.40	74.69 ± 4.80	-3.45378 to 3.82878	0.914
2nd week	74.22 ± 3.93	72.56 ± 5.67	-1.73616 to 5.06950	0.316
3rd week	72.67 ± 3.65	70.44 ± 6.16	-0.88360 to 5.32805	0.150
4th week	72.50 ± 4.18	69.22 ± 9.09	-0.72534 to 7.28089	0.102
5th week	72.94 ± 3.92	69.83 ± 7.03	0.34554 to 5.87668	0.030^∗^
6th week	72.67 ± 3.69	70.61 ± 7.20	-0.60045 to 4.71157	0.121

MD: mean difference; ^∗^*P* values: statistically significant; CI: confidence interval.

## Data Availability

All data, figures, and tables in this review paper are labeled with references.
